# Vaccination with the variable tick protein of the relapsing fever spirochete *Borrelia hermsii* protects mice from infection by tick-bite

**DOI:** 10.1186/s13071-015-1170-1

**Published:** 2015-10-21

**Authors:** Benjamin J. Krajacich, Job E. Lopez, Sandra J. Raffel, Tom G. Schwan

**Affiliations:** Present address: Department of Microbiology, Immunology & Pathology, College of Veterinary Medicine & Biomedical Sciences, Colorado State University, Fort Collins, CO USA; Departments of Pediatrics and Molecular Virology & Microbiology, Baylor College of Medicine, Houston, TX USA; Laboratory of Zoonotic Pathogens, Rocky Mountain Laboratories, National Institute of Allergy and Infectious Diseases, National Institutes of Health, 903 S. 4th Street, Hamilton, MT 59840-2932 USA

**Keywords:** *Ornithodoros hermsi*, Tick-borne relapsing fever, Spirochetosis

## Abstract

**Background:**

Tick-borne relapsing fevers of humans are caused by spirochetes that must adapt to both warm-blooded vertebrates and cold-blooded ticks. In western North America, most human cases of relapsing fever are caused by *Borrelia hermsii*, which cycles in nature between its tick vector *Ornithodoros hermsi* and small mammals such as tree squirrels and chipmunks. These spirochetes alter their outer surface by switching off one of the bloodstream-associated variable major proteins (Vmps) they produce in mammals, and replacing it with the variable tick protein (Vtp) following their acquisition by ticks. Based on this reversion to Vtp in ticks, we produced experimental vaccines comprised on this protein and tested them in mice challenged by infected ticks.

**Methods:**

The *vtp* gene from two isolates of *B. hermsii* that encoded antigenically distinct types of proteins were cloned, expressed, and the recombinant Vtp proteins were purified and used to vaccinate mice. *Ornithodoros hermsi* ticks that were infected with one of the two strains of *B. hermsii* from which the *vtp* gene originated were used to challenge mice that received one of the two Vtp vaccines or only adjuvant. Mice were then followed for infection and seroconversion.

**Results:**

The Vtp vaccines produced protective immune responses in mice challenged with *O. hermsi* ticks infected with *B. hermsii*. However, polymorphism in Vtp resulted in mice being protected only from the spirochete strain that produced the same Vtp used in the vaccine; mice challenged with spirochetes producing the antigenically different Vtp than the vaccine succumbed to infection.

**Conclusions:**

We demonstrate that by having knowledge of the phenotypic changes made by *B. hermsii* as the spirochetes are acquired by ticks from infected mammals, an effective vaccine was developed that protected mice when challenged with infected ticks. However, the Vtp vaccines only protected mice from infection when challenged with that strain producing the identical Vtp. A vaccine containing multiple Vtp types may have promise as an oral vaccine for wild mammals if applied to geographic settings such as small islands where the mammal diversity is low and the Vtp types in the *B. hermsii* population are defined.

## Background

The ability of some blood-borne pathogens to temporarily evade their host’s immune system increases the potential for these organisms to be horizontally transferred between susceptible individuals via the bite of hematophagous arthropods [1]. Such is the case with spirochetes that cause relapsing fever in humans, exemplified and best understood for *Borrelia hermsii*, the primary cause of tick-borne relapsing fever in western North America [2]. This species of bacterium is maintained in enzootic cycles that include rodent hosts and the argasid (soft) tick vector, *Ornithodoros hermsi* [3, 4]. People are accidental hosts when bitten by infected ticks while sleeping in infested cabins or when disrupting tick-infested debris [5–7].

*Borrelia hermsii* has the molecular machinery to vary antigenically [8–10]. A single organism contains the genetic potential to produce 60 or more antigenic variants, which allow the spirochetes to evade the host’s humoral immune response and sequentially produce a series of serotype-specific populations that are antigenically distinct from one another [8, 11]. These cyclic and repeated high cell densities (spirochetemias) of *B. hermsii* in a wild rodent’s blood increase the opportunity for these bacteria to be acquired by the fast-feeding ticks [12]. When humans become infected, these recurrent peaks of spirochetemia are associated with repeated episodes of acute illness, hence the name relapsing fever [13].

The large repertoire of antigenically distinct variable major proteins (Vmps) that *B. hermsii* produces on its cell surface when in the mammalian bloodstream has complicated strategies to develop sensitive and specific serological tests as well as vaccines [14]. Advancing the former need, the periplasmic enzyme glycerophosphodiester phosphodiesterase (GlpQ) in relapsing fever spirochetes is highly immunogenic and conserved among isolates; however, this protein does not produce a protective immune response [15–17]. Therefore, we tested another protein that *B. hermsii* produces, not while circulating in the bloodstream, but rather during infection in its tick vector.

When *B. hermsii* is acquired by *O. hermsi* during its blood meal, the spirochetes first accumulate in the tick’s midgut and subsequently establish persistent infections in the salivary glands and other tissues [18]. In the salivary glands, the spirochetes no longer produce the Vmp that was made in the bloodstream at the time of acquisition, but rather the spirochetes switch to making a different surface protein called the variable tick protein (Vtp) [18–21]. This protein is paralogous to the family of variable small proteins (Vsps) (a subset of the Vmps) that *B. hermsii* produces during infection in the blood [22]. However, there is only one copy of *vtp* in the *B. hermsii* genome [20, 23], and this gene is expressed by a different promoter than that by which the *vmps* are expressed [23]. Therefore, within a given clonal population or infectious lineage of *B. hermsii*, Vtp does not vary.

We hypothesized that immunization with Vtp would stimulate a protective antibody response against tick-transmitted *B. hermsii*, as does the orthologous protein, outer surface protein (Osp) C, that is produced by the Lyme disease spirochete, *Borrelia burgdorferi,* when this bacterium is transmitted by the hard tick *Ixodes scapularis* [24–28]. Like OspC in *B. burgdorferi* [29], Vtp in *B. hermsii* is polymorphic, and has seven antigenic types described to date, and the synthesis of this protein is essential for mammalian infection during transmission by tick bite [21, 30]. Therefore, we cloned and expressed the *vtp* gene from two strains of *B. hermsii* that produced different antigenic types of Vtp, immunized mice with the purified proteins, and challenged the mice with *B. hermsii* via the bite of infected *O. hermsi* ticks. Herein we demonstrate that immunization with Vtp produced a protective antibody response when the vaccinated animals were challenged with *B. hermsii* that produced the same Vtp, but not when challenged with a strain that produced a different antigenic type.

## Methods

### Bacterial isolates and cultivation

*Borrelia hermsii* isolates originated from diagnostic samples from relapsing fever patients infected in eastern Washington State (DAH) and western Montana (LAK-3). These isolates represent the two genomic groups (GG) described previously for *B. hermsii* [20, 30]. DAH is a member of GGI and LAK-3 is a member of GGII. DAH contains a *vtp* Type 6 gene while LAK-3 contains a *vtp* Type 5 gene, and the two encoded proteins excluding the signal peptide share 69.9 % amino acid identity. An isogenic mutant of *B. hermsii* DAH lacking Vtp was included in some of the experiments [21, 31]. *Borrelia hermsii* cultures were grown at 34 °C in BSK-II [32] or BSK-H medium (Sigma-Aldrich Corp., St. Louis, MO) supplemented with 12 % rabbit serum.

### Production of rabbit anti-Vtp antibody

Proteins in a whole-cell lysate of *B. hermsii* DAH passage 100 were separated by SDS-PAGE using a 17.5 % polyacrylamide gel and a preparative comb with a 14-cm continuous well. After electrophoresis, the unfixed gel was stained with water-based Coomassie Brilliant Blue lacking acetic acid, destained in water, and the dominant ~20-kDa band (Vtp) was excised with a razor blade from the gel in a 3-mm wide strip. One half of the gel strip was triturated with a sterile mortar and pestle in PBS and emulsified in an equal volume of Complete Freund’s Adjuvant while the other half of the gel was frozen at −80 °C and kept for the booster immunization. The primary immunization of the adult New Zealand white rabbit (*Oryctolagus cuniculus*) totaled 2 ml delivered by injection in four 0.5 ml doses, two intramuscularly in each of the hind legs and two subcutaneously in the nape of the neck. The rabbit received four booster shots 36 days later following the same protocol except that the gel was emulsified in Incomplete Freund’s Adjuvant. Immune serum was collected from the rabbit 35 days later.

### Production of recombinant Vtp

Total genomic DNA preparations from the two *B. hermsii* isolates were used as templates for PCR amplification of the *vtp* gene. The forward and reverse primers used to amplify the gene from both samples were 5’-TATCATATGAATAATGGAGGCCCAG-3’ and 5’-CTCGAGTCAAGG TTTAACAGGG-3’, respectively. These primers lacked the first 57 nucleotides to exclude the 19 amino acid signal peptide to optimize the heterologous synthesis of the protein in *Escherichia coli*, as Carter *et al.* reported that all attempts to clone the entire *vtp* (= *vmp33*) gene into an expression vector failed [22]. PCR amplification was performed with the GoTaq Flexi DNA polymerase kit (Promega, Madison, WI) and the DNA Engine Tetrad (Bio-Rad Laboratories, Inc., Hercules, CA). The amplification parameters included an initial heating at 96 °C for 5 min, followed by 35 cycles with a denaturing temperature of 94 °C for 30 s, an annealing temperature of 58 °C for 30 s, and an extension temperature of 72 °C for 2.5 min. Amplicons were first inserted into the pCR2.1-TOPO TA vector (Invitrogen/Life Technologies, Grand Island, NY) according to manufacturer’s instructions, and the recombinant plasmid was transformed into TOP10 electrocompetent *E. coli* cells (Invitrogen/Life Technologies). Plasmid DNA was purified with a Miniprep Kit (Qiagen, Valencia, CA) and sequenced as previously described [20],which demonstrated that no changes had been introduced in the genes.

The plasmids containing the DAH and LAK-3*vtp* genes and the pET15b expression plasmid (Novagen, Darmstadt, Germany) were digested for 2 h at 37 °C with *Nde*I and *Xho*I (New England Biolabs, Ipswich, MA). The *vtp-*containing fragments were gel-purified and ligated into pET15b with an overnight incubation at 15 °C, and the ligation products were electroporated into *E. coli* BL21 Star DE3 cells. The transformants were plated on Luria-Bertani agar plates containing carbenicillin (100 μg/ml) and single clones were screened by PCR. Positive clones were grown in Luria-Bertani broth containing carbenicillin (100 μg/ml) and expression was induced with 1 mM IPTG (isopropyl-β-D-thiogalactopyranoside) for 5 h at 37 °C. The *E. coli* lysates were examined by SDS-PAGE and immunoblots for the presence of a HIS-tagged fusion protein of the expected size. Membrane-bound proteins were probed with anti-polyhistidine horseradish peroxidase-conjugated antibody (Sigma-Aldrich Corp.) diluted 1:2,000. The proteins were purified with a Ni-NTA His-Bind resin column (Novagen) with 6 M urea-denaturing conditions, and then dialyzed in a 10 K MWCO Slide-A-Lyzer cassette (Thermo Scientific, Rockford, IL) to remove the urea. Protein concentrations were determined using the colorimetric Bio-Rad Protein Assay (Bio-Rad Laboratories) following the manufacturer’s instructions.

### Immunizations with rVtp

RML mice (*Mus musculus*) used in this study are an outbred strain that originated from the Swiss-Webster background and are bred at the Rocky Mountain Laboratories (RML). Groups of five adult mice, 6 – 8 weeks old, received their primary immunization by intraperitoneal injection that included 200 pmol of purified rVtp protein (4.45 or 4.48 μg of Vtp Type 5 and 6, respectively). Our vaccine doses (~4.5 μg) were within the range of most other recombinant protein vaccines (1 – 20 μg) used experimentally for rodents and Lyme disease spirochetes [26, 33–36]. The purified proteins were suspended in 200 μl phosphate buffered saline (PBS) and mixed with 200 μl of the MPL + TDM + CWS Adjuvant System M6661 (Sigma-Aldrich Corp.). The mice received two identical booster immunizations 28 and 56 days later. Five negative control mice for each experiment were also sham immunized and boosted twice on the same schedule with only 200 μl of adjuvant mixed with 200 μl of PBS.

### Agglutination and borreliacidal assays

Serum from the immunized rabbit was tested for its ability to agglutinate *B. hermsii* DAH cells. In a sterile 96-well flat-bottom tissue culture plate, 100 μl of serial two-fold dilutions of immune and normal rabbit serum were mixed with 100 μl of a stationary-phase (~10^8^ spirochetes/ml), high passage spirochete culture. The final serum dilutions of the suspensions were 1:16 – 1:2048, and the mixtures were incubated for 5 h at 37 °C and then kept at RT overnight. The following morning, the wells were examined with aninverted Nikon Diaphot Phase Contrast microscope (Nikon Instruments Inc., Melville, NY) to assess the presence of agglutinated spirochetes.

Serum from the Vtp-immunized rabbit was tested for borreliacidal activity along with serum from a normal non-immunized rabbit. The first assay tested the immune and control sera at a 1:2 dilution by mixing 100 μl of a stationary-phase (~10^8^ spirochetes/ml), high passage culture of *B. hermsii* DAH with 100 μl of the undiluted immune or control serum, incubating the mixtures at 37 °C for 6 h, transferring the 200 μl into tubes containing 9 ml of BSK-II medium, and incubating the cultures for 9 days at 34 °C. In a second experiment, one ml of a stationary-phase, high passage, Vtp-positive culture of *B. hermsii* DAH was mixed with 1 ml of either the immune or control serum in a sterile culture tube and placed at 37 °C. Starting 30 min later, and then every 30 min until 240 min had elapsed, 100 μl of each spirochete-serum mixture was transferred to a tube containing 9 ml of BSK-II medium. These 16 closed tubes were incubated at 34 °C and examined on days 3 and 24 for the presence of live spirochetes.

Serum samples from the immunized mice were tested for their ability to agglutinate and kill *B. hermsii*. Initially, 100 μl of a stationary phase spirochete culture was mixed with 100 μl of two-fold serial dilutions of serum (1:16 to 1:2048) collected from mice just prior to their challenge with infected ticks. These mixtures were incubated for one hr at 37 °C, kept overnight at RT, and examined with an inverted Nikon Diaphot Phase Contrast microscope.

To test viability, 50 μl of the overnight serum-spirochete suspension was mixed with 150 μl of fresh BSK-H and incubated for 7 h at 37 °C. From this mixture, 5 μl was imaged as a wet preparation slide by dark-field microscopy. These tests were repeated with serum heated to 56 °C for 30 min to inactivate complement.

### Challenge of immunized mice with *B. hermsii*-infected ticks

The *O. hermsi* SIS ticks used for these studies came from an uninfected colony maintained at the RML and originated from a single pair of uninfected adults collected from Siskiyou County, California [12]. Two cohorts of nymphal ticks were first infected with either *B. hermsii* DAH (that produced Vtp6) or *B. hermsii* LAK-3 (that produced Vtp5). The ticks were infected by feeding them on a spirochetemic mouse that had been inoculated intraperitoneally 3 – 4 days previously with a BSK-H culture that contained one of the two strains of spirochetes. Immediately after feeding, three ticks from each group were dissected and the engorged midgut from each tick was mixed in a drop of PBS and examined with a dark-field microscope. Spirochetes were easily observed in all ticks examined, which confirmed that the ticks became infected while feeding. After the acquisition feeding, the engorged nymphal ticks were kept at 21 °C and 85 % RH until they molted to their next developmental stage. For challenge, groups of 5 infected ticks were placed on each experimental animal to allow them to feed. During tick feedings, the mice were anesthetized by intraperitoneal injection of pentobarbital sodium (0.5 mg/10 g body weight) (Abbott Laboratories, North Chicago, IL).

Mice immunized with DAH rVtp (Type 6) or LAK-3 rVtp (Type 5) were challenged 23 and 24 days, respectively, after receiving their second boost. Nearly all the ticks fed during the challenge experiments. On 27 of the 30 mice, all 5 ticks attached; on one mouse, 4 of the 5 ticks attached; on two mice, 3 of the 5 ticks attached. Each mouse was checked daily on days 3 to 10 after tick feeding for spirochete infection by examining blood collected from the tail vein [17]. Mice were anesthetized with isoflurane (Fluriso) (Vet ONE, MWI Veterinary Supply, Boise ID), the tip of the tail was nicked with small surgical scissors, and a ~ 5 μl drop of blood was expressed onto a glass microscope slide. The drop was covered with a 22 mm x 22 mm cover slip and examined with a Nikon Eclipse E600 dark-field microscope and 400X dry lens (Nikon Instruments Inc.). Once an animal was found to be spirochetemic, the mouse was scored as infected and not examined again. Following tick challenge, the mice were held for an additional 60 days at which time terminal blood samples were collected for serological analysis.

### Serology

SDS-PAGE and immunoblots were performed as previously described [21]. Pre- and post-immunization serum samples from the rabbit immunized with the gel-excised Vtp were tested at 1:5000 dilutions with whole-cell lysates of the wild-type *B. hermsii* DAH and the isogenic deletion mutant lacking Vtp. The Vtp Type 6 specific mAb H4825 [20, 37] was also tested with the same lysates at 1:100 dilution to confirm the specificity of the rabbit immune serum.

Mouse serum samples were collected prior to immunization (pre-bleed), 20 days after the second boost just prior to challenge (post-immunization), and 60 days after challenge by tick-bite (post-challenge). These samples were tested for antibody reactivity by immunoblot analysis and ELISA. Immunoblots included the two purified rVtps and *B. hermsii* DAH and LAK-3 whole-cell lysates in the panel of antigens. The serum samples were tested at 1:1,000 dilutions in PBS with 0.5 % Tween-20, 0.2 % weight/volume I-block (Applied Biosystems, Foster City, CA). Bound antibodies were detected with the exposure of film using Rec- protein A-HRP (1:4,000) and the ECL Chemiluminescent Substrate Kit (Invitrogen).

Serological analyses by ELISA were done as described previously [38]. Briefly, 20 ml cultures of *B. hermsii* DAH and LAK-3 were grown to stationary phase (~10^8^/ml), the cells were centrifuged (12,500 x *g*), rinsed twice in PBS, and suspended to an OD_600_ of 0.05. These cell suspensions were sonicated and the whole-cell lysates were used as antigen for analysis. Preliminary assays were done to assess the level of antibody reactivity in the sera from the different groups of mice collected at different time points, and the assays were refined to better assess the end-point titers. The samples were tested with the homologous and heterologous antigen strains. The post-immunization samples were tested at two-fold serial dilutions from 1:32,000 to 1:512,000 with the homologous strain and at three-fold serial dilutions from 1:200 to 1:128,000 with the heterologous strain. Serum samples from the negative control mice that received only the adjuvant and PBS were tested at two-fold serial dilutions from 1:200 to 1:3,200. The pre-bleed samples were used to determine the cutoff threshold for positive samples by using their mean + 3 SD of the optical density. The secondary detection antibody was peroxidase-labeled goat anti-mouse IgG (H + L chain) diluted 1:5,000 (Kirkegaard and Perry Laboratories, Inc., Gaithersburg, MD). The reciprocal titers were transformed with the log base 2, and the geometric mean titers were calculated for each group and compared with one-way ANOVA.

### Indirect Fluorescent Antibody (IFA) staining of spirochete-infected tick tissues

Salivary glands were dissected from ticks infected with one of the two strains of spirochetes, squashed on glass microscope coverslips, dried over an open flame and then fixed in acetone. These samples were incubated with one of two Vtp-specific monoclonal antibodies, H4825 for the DAH Vtp (Type 6) [37] or H3548 for the LAK-3 Vtp (Type 5) [20] as undiluted supernatants for 30 min, rinsed in PBS, and incubated with goat anti-mouse-FITC conjugated antibody (1:100 dilution) (Kirkegaard & Perry Laboratories) for 30 min. Some preparations were stained with the rabbit polyclonal anti-Vtp antibody described herein. The coverslips were rinsed, dried, mounted with glycerol on glass microscope slides and examined with a Nikon Eclipse E800 epifluorescence microscope (Nikon Instruments Inc.).

### Ethics statement

The Rocky Mountain Laboratories, NIAID, NIH, Animal Care and Use Committee approved study protocols for the feeding of ticks on mice, mouse immunization and infection of relapsing fever spirochetes (#2011-049), and the immunization of rabbits (#93-10.54). All work was conducted adhering to the institution’s guidelines for animal husbandry, and followed the guidelines and basic principals in the Public Health Service Policy on Humane Care and Use of Laboratory Animals, and the Guide for the Care and Use of Laboratory Animals, United States Institute of Laboratory Animal Resources, National Research Council.

## Results

### Rabbit immune serum is specific for Vtp

**Fig. 1:**
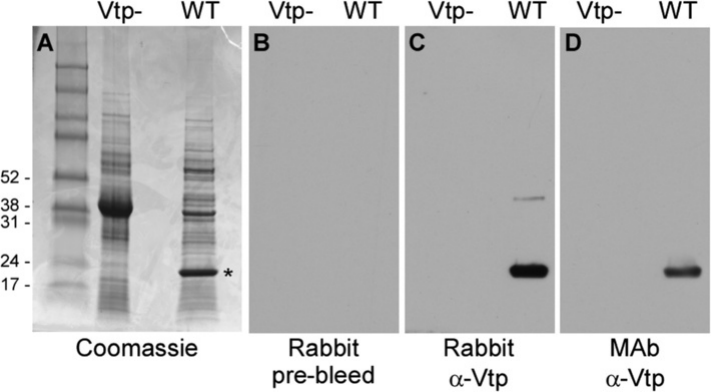
Coomassie-stained gel (**a**) and immunoblots showing the seroconversion and specificity of the immune rabbit serum to Vtp (**b**, **c**), and the recognition of Vtp Type 6 with the specific monoclonal antibody H4825 (**d**). Vtp- = *B. hermsii* mutant lacking Vtp; WT = wild type DAH producing Vtp; MAb = monoclonal antibody. Molecular mass standards on left in kDa. * Identifies Vtp (**a**)

The rabbit immunized with the excised gel fragment containing the ~20 kDa protein seroconverted to Vtp. The specificity was demonstrated by immunoblot when the pre- and post-immunization serum samples were tested with *B. hermsii* lysates of strains containing and lacking Vtp (Fig. 1). The rabbit immune serum and Vtp-specific monoclonal antibody recognized the same protein in the wild-type *B. hermsii* but nothing in the deletion mutant lacking Vtp. These results demonstrated that the rabbit immune serum was specific for Vtp.

### Rabbit anti-Vtp immune serum agglutinates and kills spirochetes

The rabbit immune serum agglutinated *B. hermsii* DAH cells at dilutions of 1:16 –1:512 (Table 1). The pattern of clumping varied from a continuous lattice at the lowest dilutions to significant, isolated clumps of cells at the middle dilutions, to no longer being detectable at the two most diluted samples (data not shown). No agglutination was observed in any of the dilution wells containing serum from the non-immunized rabbit.

**Table 1:** Agglutination of *Borrelia hermsii* DAH with rabbit anti-Vtp immune serum

	Dilution of serum tested							
Serum	1:16	1:32	1:64	1:128	1:256	1:512	1:1024	1:2048
Rabbit anti-Vtp6	+	+	+	+	+	+	-	-
Rabbit Neg. control	-	-	-	-	-	-	-	-

+, agglutination;-,no agglutination

Serum samples from the immunized and non-immunized rabbits were tested for their ability to kill *B. hermsii*. In the first assay with spirochetes exposed at a 1:2 serum dilution for 6 h at 37 °C, no spirochetes exposed to the immune serum grew when transferred into fresh BSK-II medium. In contrast, spirochetes mixed with the serum from the non-immunized rabbit grew to a high cell density in fresh medium. The second experiment with spirochetes exposed to immune or non-immune rabbit serum for 30–240 min also demonstrated that no spirochetes grew when exposed to the immune serum for as little as 30 min and then transferred to fresh medium. However, all cultures of spirochetes exposed to serum from the non-immunized rabbit grew out to high cell densities (data not shown). These initial results demonstrated that the anti-Vtp immune serum produced in the rabbit agglutinated and killed spirochetes, which led us to produce recombinant Vtps for use as experimental vaccines against *B. hermsii*.

### Immunized mice seroconvert to *B. hermsii* rVtp

**Fig. 2:**
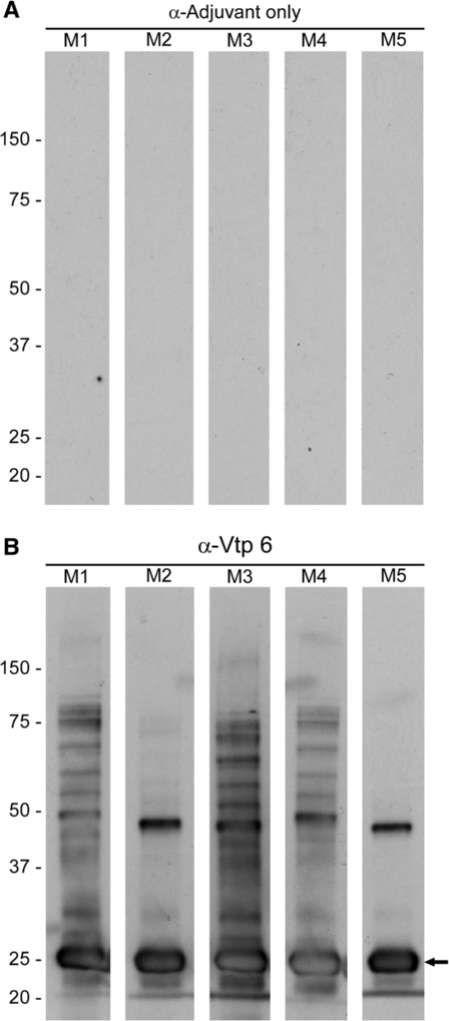
Immunoblot analysis of serum from mice immunized with only the adjuvant (**a**) or purified recombinant Vtp 6 with adjuvant (**b**). Antigen was purified Vtp 6 and serum samples were tested at 1:200 dilutions. Five mice were examined in each group (M1 – M5). Molecular mass standards on left in kDa. Arrow indicates the primary Vtp protein, slightly larger than the native protein because of the 6-histidine tag; larger aggregates of the protein are above

All mice immunized with three injections of purified rVtphad IgG antibody titers greater than or equal to 1:512,000 when tested by ELISA with the *B. hermsii* isolate producing the homologous Vtp (Table 2). Antibody titers to the heterologous strain were much lower and ranged from 1:200 up to 1:128,000 for two samples, but demonstrated varying degrees of cross-reactivity. All 10 mice immunized with only the adjuvant were either non-reactive (<1:200) or reactive at the lowest three dilutions. The same serum samples from the immunized mice demonstrated a strong antibody response by immunoblot analysis to the purified rVtp (Fig. 2).

**Table 2:** ELISA titers in mice immunized with rVtp5, rVtp6 or only adjuvant when tested with *Borrelia hermsii* whole-cell lysates DAH or LAK-3

Tested with DAH whole-cell lysate		
Vaccine	Number of Mice	Titers*
Adjuvant only	(*N* = 5)	all <1:200
Vtp6 (DAH)	(*N* = 5)	all ≥ 1:512,000
Vtp5 (LAK-3)	(*N* = 5)	1:200; 3 @ 1:5.400; ≥ 1:16,200
Tested to LAK-3 whole-cell lysate		
Vaccine	Number of Mice	Titers**
Adjuvant only	(*N* = 5)	2 @ < 1:200; 1:200; 1:400; 1:800
Vtp6 (DAH)	(*N* = 5)	1:16,000; 2 @ 1:64,000; 2 @ 1:128,000
Vtp5 (LAK-3)	(*N* = 5)	all ≥ 1:512,000

Geometric mean titers of each group significantly different, *p = 0.0008; **p = 0.0001

### Mouse immune serum to rVtp6 agglutinates and kills spirochetes

**Fig. 3:**
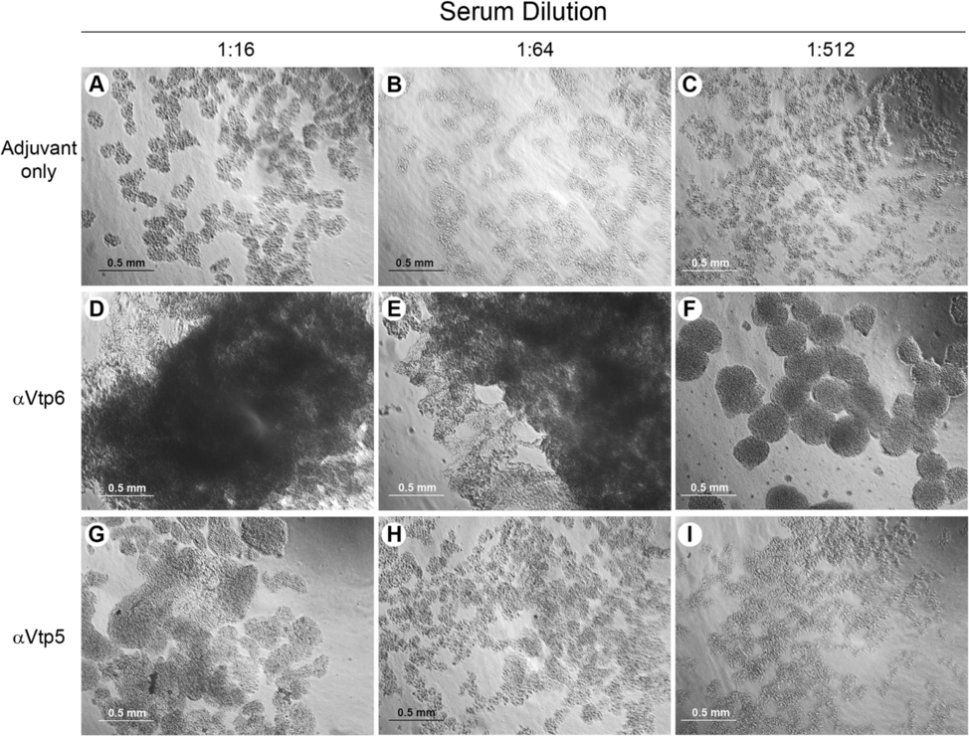
Agglutination assays with mouse immune sera and *B. hermsii* DAH cells producing Vtp Type 6. Serum from mice immunized with rVtp Type 6 caused significant aggregation of spirochetes (**d**, **e**, **f**) not observed with the serum from the mice immunized with only the adjuvant (**a**, **b**, **c**) or the heterologous rVtp Type 5 (**g**, **h**, **i**). Serum dilutions are shown on the top and the type of immune serum is shown on the left

Serum samples from mice immunized with rVtps or the adjuvant were tested for their ability to agglutinate and kill spirochetes. Serum from those mice immunized with DAH rVtp (Type 6) produced large opaque clumps of agglutinated *B. hermsii* DAH cells at 1:16 to 1:512 dilutions (Fig. 3d, e, f), whereas the serum from the heterologous-immunized and control mice did not. Unexpectedly, the immune serum from those mice immunized with LAK-3 rVtp (Type 5) failed to agglutinate *B. hermsii* LAK-3 cells. Additionally, serum from those mice immunized with DAH rVtp (Type 6) killed *B. hermsii* DAH spirochetes when diluted out to 1:128 (Table 3), which also happened with serum samples heated to inactivate complement. But serum from mice immunized with LAK-3 rVtp (Type 5) showed no borreliacidal activity when tested with *B. hermsii* LAK-3 (data not shown). We examined a lysate of the freshly grown LAK-3 strain and found that the culture had not entirely switched to producing Vtp, but rather was still making one of the variable large proteins (Vlps) that we did not attempt to identify. We continued to cultivate these spirochetes for two months, yet the population still failed to switch to only making Vtp. Therefore, we abandoned this part of the work and concluded that the immune serum produced to rVtp5 failed to agglutinate and kill spirochetes *in vitro* because the majority of the population of spirochetes used in the assay was not producing the target Vtp.

**Table 3:** Killing activity of mouse immune serum to *Borrelia hermsii* DAH (Vtp6)

	Dilution of serum tested							
Serum	1:16	1:32	1:64	1:128	1:256	1:512	1:1024	1:2048
Mouse anti-Vtp6	-	-	-	-	+	+	+	+
Mouse anti-Vtp5	+	+	+	+	+	+	+	+
Mouse Adj. Only	+	+	+	+	+	+	+	+

Serum from one mouse used from each of the three groups shown; − no growth; + growth

### Mice immunized with rVtp are protected from infection when challenged by tick bite

Cohorts of *O. hermsi* ticks infected with *B. hermsii* DAH or LAK-3 were fed on mice immunized with rVtp6, rVtp5, or only the adjuvant to assess the efficacy of the two vaccines. All mice immunized with rVtp6 or rVtp5 were protected when fed upon by ticks infected with the strain producing the same Vtp (Table 4), as no spirochetes were detected for up to10 days following challenge. In contrast, immunization with the rVtps failed to protect mice from infection when challenged by ticks infected with the strain of *B. hermsii* producing the heterologous Vtp type. Also, none of the 10 mice that received only the adjuvant were protected and became spirochetemic by day-3 post challenge (Table 4). During the heterologous challenges, one of the five mice immunized with rVtp6 failed to become spirochetemic after the first feeding of ticks infected with *B. hermsii* LAK-3. This mouse was challenged again 28 days later with 5 LAK-3 infected ticks, which resulted in infection with a detectable spirochetemia four days later.

**Table 4:** Mice immunized with rVtp are protected from infection by tick bite only when challenged with *Borrelia hermsii* producing the same Vtp antigenic type

Challenge strain	Vaccine Type	No. Infected / No. Total
*B. hermsii* DAH	Adjuvant Only	5/5
	rVtp DAH	0/5*
	rVtp LAK-3	5/5
*B. hermsii* LAK-3	Adjuvant Only	5/5
	rVtp DAH	5/5**
	rVtp LAK-3	0/5*

*Significantly different, Fisher’s exact test, *p* = 0.0003

**One mouse infected during second challenge

**Fig. 4:**
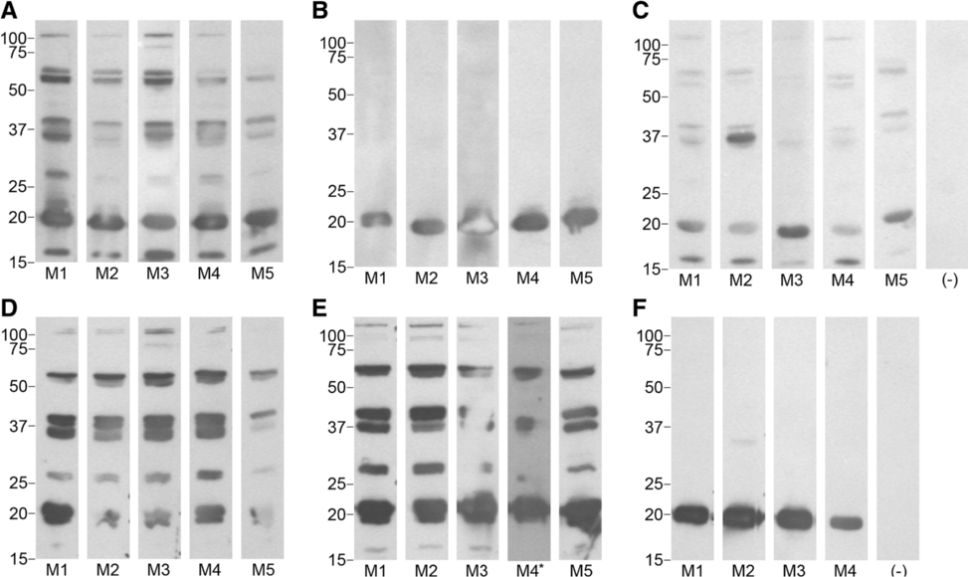
Immunoblot analysis of the serological responses in mice following immunization and challenge by tick bite. Whole-cell lysates of *B. hermsii* DAH (panels **a**-**c**) and *B. hermsii* LAK-3 (panels **d**-**f**) were tested with individual mouse serum samples (M1-M5) from each of the 6 groups of mice immunized with rVtp Type 5, rVtp Type 6, or only the adjuvant. Mice challenged with the strain producing the same Vtp as the vaccine were protected from infection, and showed no expansion of antibodies after challenge other than to the vaccine (the ~20 kDa protein) (panels **b** and **f**). Mice immunized with the heterologous rVtp (panels **c** and **e**) or only the adjuvant (panels **a** and **d**) became infected and showed an expanded antibody response after challenge, although the response was weaker in some animals (panel **c**). One mouse in the LAK-3 homologous challenge group (panel **f**) died before the final serum samples were collected. * indicates the mouse that was infected from the second challenge. (−) shows serum samples from non-immunized, uninfected mice. Molecular mass standards are shown in kDa

Serum samples collected from the mice 60 days after being fed upon by infected ticks (post challenge) were tested by immunoblot with whole-cell lysates of the homologous strain. Those animals immunized with rVtp and challenged with the homologous strain (mice protected from infection) produced antibodies only to the native Vtp (the vaccine) (Fig. 4b, f). In contrast, serum samples from animals challenged with the heterologous strain(Fig. 4c, e), and those mice that received only the adjuvant (Fig. 4a, d), showed reactivity to multiple proteins demonstrating seroconversion as a result of infection, although the strength and diversity of the responses varied among the groups. Together, both the monitoring of spirochetemia and the serological analysis demonstrated that immunization with rVtp conferred protection from *B. hermsii* infection when the vaccinated mice were fed upon by infected ticks harboring the strain producing the identical Vtp but not with a Vtp of an antigenically different type.

### Demonstration of spirochetes in ticks used to challenge vaccinated mice

None of the 10 immunized mice that were challenged with *B. hermsii* producing the homologous Vtp became infected. To exclude the possibility that this result might be due to the challenge ticks not being infected, we fed the surviving members of each tick group again two months later on non-immunized mice. Nine of the 10 original tick groups (4 of the 5 groups infected with DAH; all 5 groups infected with LAK-3) had 2 – 5 surviving ticks, and when each group was fed on a non-immunized mouse, all 9 animals became infected with a detectable spirochetemia 3–4 days later.

**Fig. 5:**
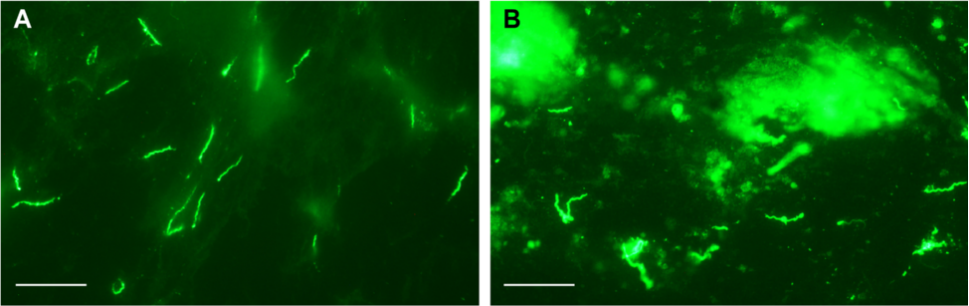
*Borrelia hermsii* LAK-3 in a tick salivary gland following tick feeding on an immunized mouse (**a**) and a control mouse receiving only the adjuvant (**b**). IFA stain with Vtp Type 5 specific monoclonal antibody H3548 (**a**) and a rabbit polyclonal anti-Vtp antibody (**b**). Scale bars represent 20 μm

Additionally, three ticks from one group infected with DAH and one group infected with LAK-3 were examined microscopically for infection after feeding first on the vaccinated mice and then on the non-immunized mice. IFA stains were performed on the tick salivary glands using monoclonal antibodies specific for each Vtp. All six ticks were infected and contained spirochetes in the salivary glands that were Vtp-positive (Fig. 5a). Thus the lack of infection in those immunized mice fed upon by ticks infected with *B. hermsii* producing the homologous Vtp was not due to the challenge ticks being uninfected, but rather through the protection afforded by the vaccination. As expected, infected ticks that fed on the control mice that were immunized with only the adjuvant also contained spirochetes in the salivary glands that produced Vtp (Fig. 5b).

## Discussion and conclusions

Due to the ability of relapsing fever spirochetes to evade the host’s humoral immune response through varying the antigenic composition of their major outer surface proteins, the immunological responses to infection have received much attention [37, 39–42]. In spite of this, little effort has focused on vaccines for these infections or developing experimental strategies for testing such applications in the laboratory. This is in stark contrast to efforts devoted to developing a vaccine for Lyme disease [43, 44], which is another tick-transmitted zoonotic spirochetosis [45–47]. The lack of interest in developing a vaccine for tick-borne relapsing fever has likely been due, in part, to the complex and ephemeral nature of the relapsing fever spirochete’s changing antigenic surface [37, 48], which has been viewed as a major obstacle toward the development of a vaccine [49].

Interest in vaccines for Lyme disease arose soon after the discovery of the cause of this illness was reported [45]. Johnson and colleagues first demonstrated that hamsters could be passively and actively immunized to protect these animals from infection when challenged by needle inoculation with culture-derived *B. burgdorferi* [50, 51]. Active immunity in these animals was produced with a killed, lyophilized whole-cell spirochete preparation [50]. This “bacterin” whole-cell vaccine was soon developed into a commercial vaccine for dogs, which is still in use today [43, 52].

Early studies of the protein composition of Lyme disease spirochetes identified a major outer surface protein (Osp) of approximately 31,000 MW [53], which was subsequently named OspA [54]. Several studies demonstrated that both monoclonal antibodies and monospecific polyclonal immune serum produced to OspA passively protected SCID and immunocompetent mice from clinical disease and infection with *B. burgdorferi* [33, 34, 55]. Fikrig and coworkers demonstrated that active immunization of immunocompetent mice with purified recombinant OspA produced a protective antibody response [33], results which were confirmed by other investigators [26, 56]. These corroborating results included the use of European strains of the spirochete and challenge with infected ticks [36, 57]. The mechanism of protection for anti-OspA antibodies was based on the borreliacidal activity of the immune serum when imbibed by infected ticks when they fed. The protein-specific immune serum killed the OspA-producing borrelia in the midgut of the ticks prior to the spirochetes’ dissemination and transmission via the salivary glands [35, 58].

A second major outer surface protein to receive much attention while characterizing these spirochetes was a dominant 22,000 MW, immunogenic protein first named pC [59], and later renamed OspC [60]. Numerous studies demonstrated that both passive and active immunizations involving specific immune serum or this protein conferred protection when the immunized animals were challenged by either needle inoculation or by the bite of infected ticks [25, 26, 56, 61, 62], however, protection was strain-specific [24, 56]. The specificity for protection was based on the amino acid composition of OspC, which is polymorphic among *B. burgdorferi* populations [29, 63–69]. Many serotypes of OspC of *B. burgdorferi sensu lato* have been described, with amino acid sequences within each type being identical or nearly so, while sequences between different types share only 62–80 % identity [29, 63, 66]. Thus vaccination with OspC is only protective when the immunized host is challenged with spirochetes that produce the same OspC serotype, which is what we demonstrate here with our Vtp vaccines for *B. hermsii*.

*B. hermsii* Vtp and *B. burgdorferi* OspC are orthologous proteins [20, 22, 70] and essential for mammalian infection when these spirochetes are transmitted by tick bite [21, 27, 71]. While OspC stimulates an early antibody response in the majority of Lyme disease patients [72], the humoral immune response to Vtp in patients infected with *B. hermsii* has not been evaluated. After transmission by tick bite, *B. hermsii* switches off Vtp, producing instead a bloodstream Vmp [18]. Yet the polymorphism of Vtp, as with OspC, suggests that the sequence diversity is driven and balanced by immune pressure from the natural vertebrate host populations [29, 73].

Our interest in pursuing an experimental Vtp vaccine was stimulated in part by 1) the efficacy of the OspC vaccines for *B. burgdorferi* when tested in homologous challenges, 2) our previous findings that *B. hermsii* produces Vtp and not one of the many bloodstream Vmps during transmission by tick bite, and 3) by our initial observations presented here that immune serum produced in a rabbit to gel-excised Vtp agglutinated and killed Vtp-producing *B. hermsii in vitro*. Early investigations demonstrated that immune serum made to several species of relapsing fever spirochetes agglutinated those cells to which the antibody-containing sera were produced [74]. Studies with *B. hermsii* showed the ability of various immune sera to agglutinate, immobilize, or kill spirochetes [75–78]. Most relevant to our agglutination results was the demonstration that the anti-Vtp specific monoclonal antibody H4825 [75] agglutinated *B. hermsii* cells producing Vtp [77]. Also, two IgM monoclonal antibodies produced to serotype 7 *B. hermsii* cells agglutinated spirochetes and inhibited their growth *in vitro* [76]. Mouse ascetic fluid containing IgG antibodies to an unidentified *B. hermsii* surface protein of approximately 22 kDa (possibly Vtp) also inhibited growth and killed spirochetes [79].

In addition to causing agglutination and inhibiting spirochete growth, immune sera may passively protect animals from infection with *B. hermsii* [76, 80] and other tick-borne species of spirochetes such as *Borrelia duttonii* [81, 82]. Stoenner and colleagues tested a serotype 7-specific polyclonal antibody to passively protect mice from infection with *B. hermsii* serotype 7 cells [80]. When the recipient mice were inoculated with an *in vitro* culture of serotype 7 cells that also contained small numbers of variant spirochetes, only the variants grew out; no serotype 7 cells were detected in the mice. Barbour and Bundoc also showed that mice were protected from infection with *B. hermsii* serotype 7 cells when passively immunized with anti-serotype 7 IgM monoclonal antibodies [76], yet active immunization of mice with purified recombinant Vlp7 protein failed to protect mice from infection when challenged with serotype 7 spirochetes. In fact, while many studies have reported that infections with relapsing fever spirochetes produce some level of immunity and protection from re-infection [74, 83–85], we are unaware of any studies that demonstrate protection to infection produced by active immunization with a single protein or subunit vaccine for relapsing fever spirochetes.

Lyme disease vaccines have also targeted white-footed mice (*Peromyscus leucopus*), a primary reservoir in the wild, to break the transmission cycle and thereby reduce the risk of infection to humans [86, 87]. The notion of reducing the prevalence of *O. hermsi* ticks infected with *B. hermsii* in the wild by immunizing the vertebrate hosts with a Vtp-based oral vaccine is intriguing but fraught with potential difficulties. First, the white-footed mice need to be protected from infection with *B. burgdorferi* only when the new generation of infected *I. scapularis* nymphs feeds on them. Unfed larvae, which feed on white-footed mice too but are not infected transovarially [88], and adult ticks that feed on much larger mammals like deer [47], play no direct role in the chain of infection [89]. In striking contrast, every active stage of *O. hermsi* has the potential to transmit *B. hermsii* when they feed on their hosts, primarily squirrels and chipmunks (*Tamiasciurus* spp and *Tamias* spp) [90, 91]. Larvae may become infected transovarially [92, 93], although the frequency of this event needs further study. The three or occasionally four nymphal stages are all competent vectors, as are the adults, which can feed and transmit spirochetes repeatedly [94] (unpublished results).

In the laboratory, white-footed mice infected with *B. burgdorferi* and subsequently vaccinated orally with OspA reduce the prevalence of infected *I. scapularis* nymphs that fed on them as larvae [95]. When the uninfected larvae ingest anti-OspA immune serum with the spirochetes that upregulate OspA soon after their acquisition [96, 97], the spirochetes are killed in the tick’s midgut [35]. The potential for simultaneous acquisition of spirochetes and immune serum by ticks feeding on vaccinated mice in the wild is possible because white-footed mice, once infected, remain so for life. However, the duration of infection of *B. hermsii* in mammals such as chipmunks is transient in comparison and lasts only a few weeks [98]. While immunization of chipmunks and squirrels in the field should protect these animals from infection with *B. hermsii* producing the homologous Vtp when fed upon by infected ticks, the immune serum would likely have no impact on the prevalence of infected ticks. If *O. hermsi* ticks were to acquire spirochetes from an immunized host, the spirochetes would not be producing Vtp at the time of acquisition [21], and therefore not be susceptible to killing by the immune serum. Also, given that *B. hermsii* persists in the tick’s salivary glands rather than the midgut as does *B. burgorferi*, those spirochetes producing Vtp in the salivary glands may not be subjected to immune clearance when ticks ingest blood containing anti-Vtp antibodies. This notion was supported by our observations in which infected *O. hermsi* ticks that fed on mice immunized with the homologous Vtp remained infected, retained Vtp-producing spirochetes in their salivary glands, and subsequently infected naïve mice. These observations, and the rapidity at which relapsing fever spirochetes may be transmitted after tick attachment (less than 1 min) [99, 100], indicate that these Vtp vaccines result in the killing of spirochetes in the immunized host rather than blocking transmission by killing spirochetes in ticks prior to transmission.

If oral immunization with Vtp were to produce a protective immune response (yet to be determined), a field application providing the best chance to immunize a significant proportion of wild hosts might be an island community that has a small and isolated geographic area, a reduced diversity of host species, and a limited diversity of Vtp types in the *B. hermsii* population. Such potential target areas exist on islands in Flathead Lake of western Montana [101], where tick-borne relapsing fever caused by *B. hermsii* is endemic [5, 30]. Here, baited stations similar to those developed for the control of fleas during epizootics of plague [102] could be applied to attract pine squirrels (no chipmunks live on the islands) to provide a multivalent vaccine, such as proposed for a Lyme disease OspC vaccine [103], which contains all the Vtp types present on the island. Various approved acaricides in a dust formulation could be added to these bait stations, so the squirrels would contaminate their hair while eating the vaccine-impregnated bait, and carry the toxic dusts to their nests to better target the ticks. A persistent, multiannual application could reduce the population of infected ticks and thereby lessen the risk of human infection for people visiting these islands. Current efforts to identify salivary gland antigens and midgut membrane proteins of *Ornithodoros* species of ticks as potential anti-tick vaccines might also hold promise in the future to help reduce targeted tick populations in discrete natural foci of infection [104].

Our development of the experimental vaccine presented here was based on knowledge of how *B. hermsii* adapts and changes its outer surface when persistently infecting the tick vector’s salivary glands [18, 21]. Such phenotypic switches may occur in other species, such as *B. duttonii* and *Borrelia recurrentis*, which cycle directly between human hosts with their tick and louse vectors, respectively, without involvement of wild animal reservoirs. Future investigations delving into the mechanisms of how these species alter their outer surface while infecting their arthropod vectors could provide new strategies for protection in highly endemic areas.
